# Successful salvage therapy with glofitamab followed by selinexor maintenance in an elderly patient with primary refractory diffuse large b-cell lymphoma harboring *TP53* and *CD58* mutations: a case report and literature review

**DOI:** 10.3389/fonc.2026.1908174

**Published:** 2026-10-28

**Authors:** Yurong Zhang, Huan Liu, Liangliang Li, Chune Wang, Jiajia Cao, Pengyun Zeng, Lingling Yue, Chongyang Wu

**Affiliations:** 1 Lanzhou University Second Hospital, Lanzhou, China; 2 Lanzhou University, Lanzhou, China

**Keywords:** *TP53* mutation, *CD58* mutation, glofitamab, selinexor, relapsed/refractory diffuse large B-cell lymphoma (R/R DLBCL), TFR

## Abstract

Diffuse large B-cell lymphoma (DLBCL) is the most common aggressive B-cell lymphoma characterized by pronounced heterogeneity. Patients with relapsed/refractory (R/R) disease after standard R-CHOP regimen face an extremely poor prognosis with subsequent salvage lines. Although chimeric antigen receptor T-cell (CAR-T) therapy represents a breakthrough option for R/R DLBCL, its clinical utility is frequently constrained by various real-world factors in elderly patients. As an alternative T-cell-engaging modality, CD3×CD20 bispecific antibodies (BsAbs) have demonstrated robust efficacy in R/R DLBCL. Herein, we report a 70-year-old male with primary refractory DLBCL harboring co-existing *TP53* and *CD58* mutations. The patient was refractory to prior lines of therapy, including R-CDOP(rituximab, cyclophosphamide, liposomal doxorubicin, vincristine, and prednisone), R-GemOx(rituximab, gemcitabine, and oxaliplatin), a BTK inhibitor (zanubrutinib, 160 mg bid), and Pola-BR regimen (polatuzumab vedotin, bendamustine, and rituximab). Remarkably, he achieved a sustained CR following 12 cycles of glofitamab monotherapy and subsequent the XPO1 inhibitor selinexor maintenance, complicated solely by a manageable tumor flare reaction (TFR). This case suggests that glofitamab, potentially followed by targeted maintenance, offers a promising, highly effective, and manageable treatment strategy for elderly patients with ultra-high-risk, multi-line refractory DLBCL.

## Introduction

DLBCL represents the most prevalent aggressive mature B-cell non-Hodgkin lymphoma in adults. Although the standard frontline immunochemotherapy, exemplified by the R-CHOP regimen, has dramatically improved patient survival, approximately 35% to 40% of patients experience primary refractory disease or subsequent relapse (R/R DLBCL) (1). Mutations in the *TP53* gene occur in 9% to 36% of DLBCL cases (2), constituting one of the most frequent genetic alterations and an established independent indicator of dismal prognosis. *TP53* mutations in DLBCL simultaneously abolish wild-type p53 function and confer gain-of-function activities on the mutant protein, driving genomic instability, metabolic reprogramming, IFN response repression, T cell exhaustion, and immunosuppressive TME remodeling. Concurrent *CD58* loss-of-function disrupts CD58–CD2 co-stimulation. Together, these alterations promote immune escape, sustained proliferation, and therapeutic resistance, culminating in dismal outcomes (3–5). *TP53* mutations commonly confer resistance to conventional chemotherapeutic agents (3, 4, 6, 7), and traditional salvage strategies—including high-dose chemotherapy followed by autologous stem cell transplantation (ASCT)—frequently fail to overcome the adverse prognostic impact of these alterations (8). Management options are severely restricted for elderly patients with R/R DLBCL. Although CAR-T cell therapy has emerged as a revolutionary modality, its widespread clinical implementation is frequently hampered by logistical constraints, prohibitive costs, and potential severe toxicities (9–11). CD3×CD20 BsAbs offer an alternative paradigm as an “off-the-shelf” immunotherapy, presenting a novel therapeutic avenue for this vulnerable population (12).Here, we report the case of a 70-year-old male with primary refractory DLBCL with concurrent *TP53* and *CD58* mutations. Despite failing multiple prior systemic interventions and undergoing two surgical resections. The patient achieved sustained CR with monotherapy of glofitamab and maintenance treatment with selinexor. This case aims to provide valuable insights into the management of ultra-high-risk, elderly R/R DLBCL in later-line settings.

## Case presentation

A 70-year-old male patient (height: 182 cm, weight: 66 kg) with a medical history of type 2 diabetes mellitus presented to a specialized oncology hospital in Shanghai in June 2023. Physical examination revealed a fixed, non-tender palpable mass measuring approximately 10 × 20 cm in the right upper abdomen. His Eastern Cooperative Oncology Group (ECOG) performance status was 0. A baseline 18F-FDG PET/CT scan performed on June 8, 2023, revealed multiple fluorodeoxyglucose (FDG)-avid lymphadenopathies involving the left neck, infradiaphragmatic regions, axilla, abdominal cavity, and retroperitoneum. The largest abdominal lesion measured approximately 6 × 5 cm with a maximum standardized uptake value (SUVmax) of 26.2, displaying poorly defined borders with the adjacent gastric wall and duodenum (**Figure 1A**). He underwent an intra-abdominal mass resection and biopsy, postoperative histopathology confirmed a diagnosis of DLBCL. Immunohistochemical (IHC) analysis demonstrated that the tumor cells were positive for CD20, CD79a, Bcl-2 (~90%), MUM1, MYC (~40%), p53 (~90%), and Ki-67 (~90%), meeting the criteria for double-expressor lymphoma (DEL; MYC ≥ 40% and Bcl-2 ≥ 50%). Collectively, the patient was diagnosed with DLBCL (Stage III, International Prognostic Index [IPI] score of 3, high-intermediate risk group).

**Figure 1:**
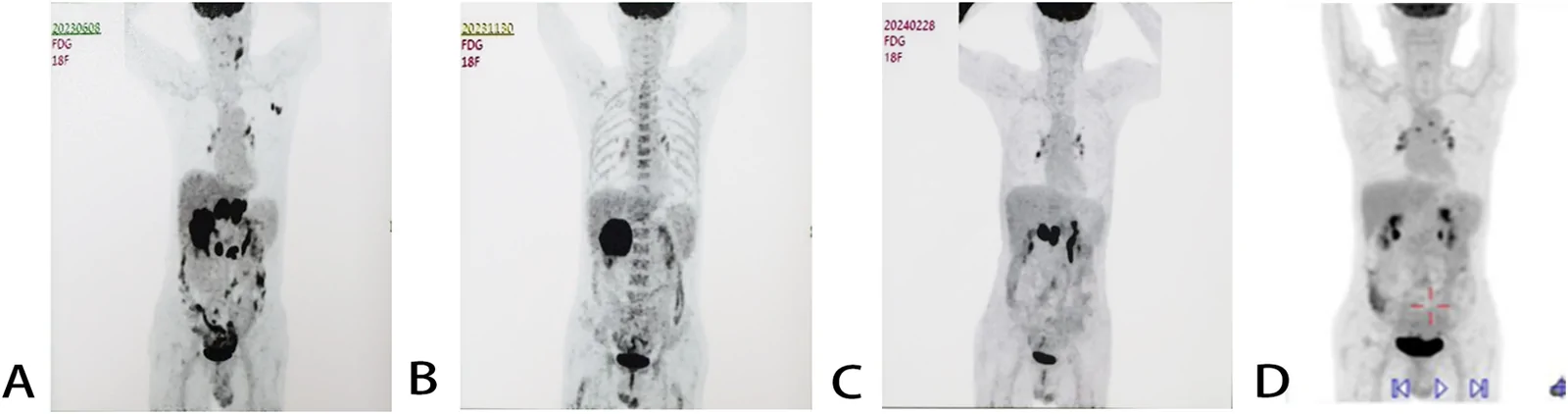
**(A)** Baseline PET-CT (2023.6.8); **(B)** PET-CT after R-CDOP regimen treatment (2023.11.30); **(C)** PET-CT after R- Gemox regimen treatment (2024.2.26); **(D)** PET-CT after Glofitamab regimen treatment (2025.7.4).

The patient subsequently received six cycles of the first-line R-CDOP regimen, administered every 3 weeks. PET/CT scan on November 30, 2023 revealed a persistent soft tissue mass beneath the gastric antrum (SUVmax 30.0) (**Figure 1B**). The treatment response was evaluated as stable disease (SD), confirming primary refractory disease.

On December 11, 2023, the patient underwent a total surgical resection of the abdominal tumor. Concurrently, next-generation sequencing (NGS) was performed on the resected tissue, revealing complex genetic alterations: (1) a missense mutation in exon 7 of the *TP53* gene with an allele frequency (AF) 30.46%; (2) a nonsense mutation in exon 3 of the *CD58* gene, AF 21.63%; (3) *BCL2* gene copy number gain; (4) *CDKN2A* gene copy number loss; and (5) *CDKN2B* gene copy number loss. The postoperative pathology was consistent with recurrent DLBCL.A second-line two cycles of R-GemOx regimen was initiated. However, a follow-up PET/CT scan on February 28, 2024, revealed a new soft tissues (3.2 cm in diameter, SUVmax 20.8) surrounding the jejunal anastomosis and the prior surgical site, indicating active lymphoma infiltration and further disease progression (**Figure 1C**).

Subsequently, a third-line targeted regimen---BTK inhibitor was administered for 4 months. A subsequent contrast-enhanced CT scan revealed persistent residual masses in the hepatic portal region. On July 4, 2024, the patient was admitted to our institution for fourth-line therapy with the Pola-BR regimen. Following four cycles, a non-contrast CT scan on September 18, 2024, demonstrated a persistent abdominal mass measuring 6.3 × 6.9 cm, which was stable compared to the pre-treatment imaging from July 2024, confirming progressive disease (PD).

Given his advanced age, DEL, high-risk *TP53* and *CD58* co-mutations, and high IHC expression of p53, the patient’s condition remained unmitigated despite multiple prior systemic therapies and surgical interventions. Diagnosed with ultra-high-risk, primary refractory elderly DLBCL, he was initiated on fifth-line glofitamab monotherapy (CD20 expression was not re-tested owing to the absence of a repeat biopsy prior to glofitamab initiation, which represents a limitation of this case). On October 14, 2024, obinutuzumab pretreatment was administered (Cycle 1, Day 1 [C1D1]) to reduce the risk of CRS. This was followed by the first step-up dose of glofitamab at 2.5 mg on October 21, 2024 (C1D8). However, A subsequent non-contrast abdominal CT scan on October 28, 2024(C1D15), confirmed that the abdominal mass had escalated to 7.0 × 8.2 cm (**Figure 2**), representing a significant increase from the baseline measurements taken on September 18, 2024. Considering the patient’s excellent systemic performance status and the absence of clinical deterioration, The patient was observed without treatment, as this transient enlargement was considered a potential TFR following the first glofitamab dose. On the same day, the second step-up dose of glofitamab (10 mg) was safely administered, and full-dose glofitamab (30 mg, every 21 days) was successfully initiated from Cycle 2 onwards. Follow-up non-contrast CT scan after Cycle 2 demonstrated a profound reduction in the size of the abdominal lesion, satisfying the criteria for a partial response (PR). Following Cycle 8 (April 1, 2025), an abdominal ultrasound revealed no detectable mass, indicating a CR. By June 2025, the patient had successfully completed the full per-protocol course of 12 cycles of glofitamab monotherapy. Throughout the dual-antibody treatment, TFR was the sole adverse event noted, which spontaneously resolved without medical intervention. To sustain the remission against the high-risk genetic background, maintenance therapy with the XPO1 inhibitor selinexor (40 mg qw) was subsequently initiated. A surveillance 18F-FDG PET/CT scan on July 4, 2025, confirmed a sustained complete metabolic response (CMR) with a Deauville score of 1 (**Figure 1D**). The time to PR was 42 days, and the time to CR was 5.5 months from the initiation of glofitamab. To date, the patient has completed over 1 year of selinexor maintenance and continues to maintain a durable CR with an excellent performance status (**Figure 3**). The treatment timeline for this patient is summarized in **Figure 3**.

**Figure 2:**
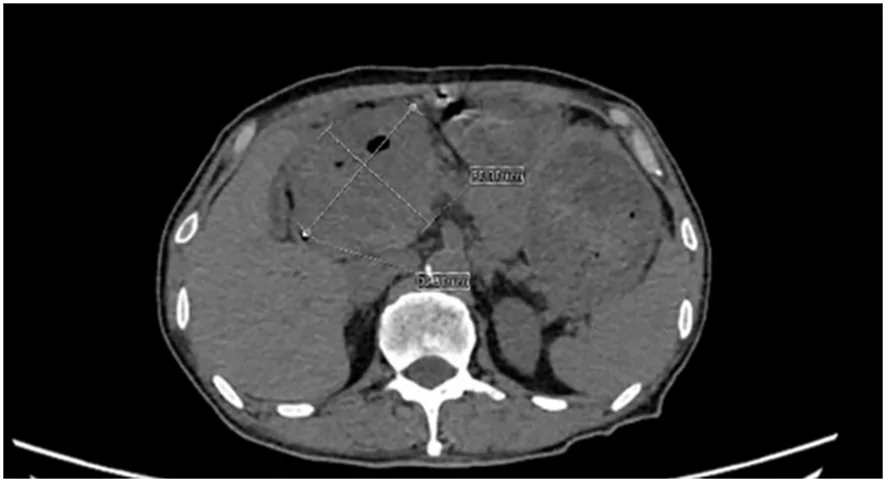
Non-contrast abdominal CT scan (2024.10.28 Glofitamab regimen C1D15) confirmed that the abdominal mass had escalated to 7.0 × 8.2 cm.

**Figure 3:**
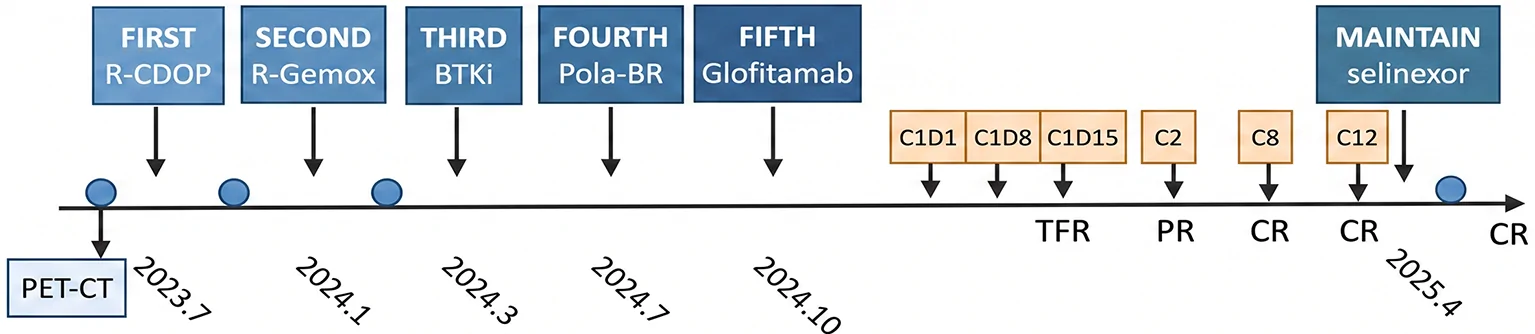
The timeline of treatment process in this case. PR, partial response; CR, complete response; TFR, tumor flare reaction.

## Discussion

Approximately 10% to 15% of patients with DLBCL experience primary refractory disease (13), The prognosis for refractory DLBCL is notoriously dismal, with median overall survival (OS) of only 5.9 to 6.3 months. Outcomes remain consistently poor in the subgroup of elderly patients aged ≥65 years (14, 15). Although modern therapeutic options—including the Pola-BR regimen, CD19-targeted monoclonal antibodies, and various small-molecule inhibitors—have enriched the salvage landscape (16–22), their long-term efficacy in the *TP53*-mutated population remains severely limited. Although CAR-T therapy shows real-world efficacy despite higher comorbidity burden and worse performance status (9, 23), *TP53*- or *CD58*-mutant patients often associated with failure after CART treatment (24, 25).

The complex genomic alterations identified in this patient collectively created an ultra-resistant oncogenic background through multi-pathway mechanisms. The concurrent *BCL2* copy number gain coupled with *CDKN2A/B* deletions conferred an anti-apoptotic and hyper-proliferative cellular state (26). Most critically, the patient harbored a hotspot missense mutation in exon 7 of the *TP53* gene (c.706T>C, p.Tyr236His) within the DNA-binding domain (DBD), which is the functional core required for p53-mediated transcriptional regulation. This specific DBD missense mutation typically induces conformational alterations in the p53 protein, abolishing its capacity to trigger cell cycle arrest and apoptosis. Growing evidence indicates that *TP53* missense mutations are important adverse prognostic factors in DLBCL, yet the impact is heterogeneous depending on the specific mutation type, structural domain, and residual function and other factors, necessitating precise molecular stratification to identify a “very high-risk” subset (3, 27–29). Immunohistochemistry revealed aberrant p53 protein overexpression in 90% of tumor cells, indicating a classic high-risk profile that accounts for this patient’s primary resistance to both first-line R-CDOP and second-line R-GemOx immunochemotherapy.

In tandem with *TP53* dysfunction, the patient harbored a deleterious *CD58* nonsense mutation. The interaction between *CD58* on tumor cells and CD2 on T cells provides a indispensable co-stimulatory signal required for host T-cell recognition and subsequent cell-mediated cytotoxicity. The *CD58* nonsense mutation (causing premature translation termination) substantially impairs host immune surveillance and blunts endogenous T-cell responses (30). Extant literature underscores that the co-existence of *TP53* and *CD58* mutations drives a synergistic “uncontrolled proliferation plus immune evasion” phenotype (31). Crucially, *CD58* loss is an established major mechanism underlying resistance or early relapse following CD19-targeted CAR-T cell therapy (25, 32, 33). Thus, patients with this dual-genetic deficit experience significantly worse outcomes even when treated with CAR-T than those with single or double wild-type profiles.

In a comprehensive review on bispecific antibody (BsAbs) resistance mechanisms in DLBCL, highlighted that genetic alterations impacting tumor-intrinsic signaling and immune surveillance represent major barriers to sustained immunotherapy responses (34). Tumor-intrinsic *TP53* mutations confer profound chemo-resistance, facilitate disease progression during BsAb therapy, and foster an immunosuppressive tumor immune microenvironment (TIME) characterized by polarization toward an M2-like “foam” macrophage phenotype, CD8+ T-cell exclusion, PD-L1 upregulation, and MHC-II downregulation (35). Concurrently, loss-of-function *CD58* alterations abolish the CD58–CD2 co-stimulatory pathway, leading to impaired T-cell synapse formation, immune escape, and failure of T-cell-directed therapies (30). Strikingly, glofitamab successfully circumvented this dual-resistance phenotype in our patient. Owing to its novel 2:1 structural format(comprising two CD20-binding domains and one CD3-binding domain), glofitamab facilitates high-avidity B-cell cross-linking and robust, direct T-cell redirection that acts independently of classical CD58–CD2 co-stimulation (25, 33). Crucially, recent evidence indicates that undetectable ctDNA after Cycle 3 predicts significantly longer median PFS (26.4 vs. 2.5 months), even in patients harboring adverse *TP53* mutations (36). These findings demonstrate that early dynamic molecular clearance can override baseline adverse genetic profiles, underscoring the potential of ctDNA monitoring for therapeutic optimization in glofitamab-based salvage strategies.

Sequential maintenance therapy with the selective inhibitor of nuclear export (XPO1) selinexor was strategically introduced to solidify complete remission in this ultra-high-risk genetic background. Clinical evidence supporting this approach stems from the pivotal SADAL phase 2b trial, which established the durable single-agent efficacy of selinexor in R/R DLBCL, with complete responders achieving a median duration of response (mDOR) of 23.0 months (19). Mechanistically, selinexor reversibly blocks XPO1 to prevent the nuclear-to-cytoplasmic transport of multiple tumor suppressor proteins, thereby restoring p53-mediated transcriptional activation and tumor suppression (37). Beyond its direct cell-intrinsic pro-apoptotic and metabolic disruption effects, ex vivo and *in vivo* studies demonstrate that selinexor profoundly reshapes the immunosuppressive microenvironment by preventing macrophage polarization toward M2 populations and mitigating macrophage-mediated T-cell inhibition (38). This microenvironmental re-sensitization acts synergistically with prior T-cell engager therapy. Regarding safety, nausea represents the primary adverse effect of selinexor. In our patient, proactive antiemetic prophylaxis—combining a 5-HT3 receptor antagonist, NK1 receptor antagonist, and low-dose daily olanzapine—effectively prevented severe gastrointestinal toxicity, enabling uninterrupted, long-term maintenance with tolerability and quality of life (39).

Another highly instructive facet of this case was the occurrence of a TFR during glofitamab treatment which was rarely detailed in the context of bispecific antibodies (40). Mechanistically, TFR typically manifests within hours to days following T-cell engagement and is pathologically characterized by the intense recruitment and infiltration of immune effector cells rather than true neoplastic proliferation (41, 42). Critically, TFR must be differentiated from true PD, it is typically self-limiting, though severe cases can be managed with low-dose corticosteroids or short-course radiotherapy without abandoning the underlying immunotherapy (42). In our case, the patient’s abdominal mass increased to 7.0 × 8.2 cm on day 7 following the first glofitamab dose from a baseline of 6.3 × 6.9 cm. However, by closely monitoring his excellent systemic performance and electing to maintain the scheduled glofitamab regimen, a rapid and dramatic tumor shrinkage was subsequently documented. This highlights that the timely and accurate recognition of TFR can effectively avert premature, erroneous treatment discontinuations.

## Patient perspective

In the early treatment phase, the treatment was fraught with challenges. Multiple surgeries and various treatment regimens yet none brought the disease to remission. There seem to be no better options apart from CAR-T and bispecific antibodies. CAR-T was unavailable for me, so I chose the bispecific antibody. During treatment with glofitamab, I did not experience any notable adverse reactions such as fever, fatigue, or changes in appetite. Although the treatment posed a certain financial burden, it remained within an acceptable range. During the first month of selinexor maintenance, I developed grade 1 nausea and reduced appetite; however, these symptoms were managed with olanzapine and aprepitant as antiemetic therapy and clearly diminished after one month. After three months, I was able to tolerate selinexor even after discontinuing antiemetic treatment, and no other adverse reactions occurred. Overall, the treatment course was much smoother than I had anticipated and had little impact on my normal life.

## Conclusion

In conclusion, *TP53* mutations constitute an established adverse prognostic factor in DLBCL, and concurrent *CD58* alterations further disrupt the tumor immune microenvironment, driving exceptionally dismal clinical outcomes. Currently, effective therapeutic strategies capable of overcoming *TP53*-mediated chemoresistance in R/R DLBCL remain profoundly restricted. While CAR-T cell therapy has remarkably improved survival in elderly R/R DLBCL, its long-term durability remains suboptimal in patients harboring *TP53* mutations or *CD58* mutations, who exhibit an inherently high risk of early relapse.

As a highly accessible, “off-the-shelf” T-cell-redirecting modality, CD3×CD20 bispecific antibodies—exemplified by glofitamab—not only offer an immediate intervention for patients ineligible for CAR-T manufacturing or those with rapidly progressive disease, but also present a viable salvage option post-CAR-T failure (43). Crucially, by virtue of its unique 2:1 structural configuration, glofitamab potently engages T cells and may substantially optimize the therapeutic response in elderly, ultra-high-risk R/R DLBCL patients with *TP53* and *CD58* mutations, while sequential selinexor therapy maintained long-term remission. Nevertheless, available data addressing this specific cohort remain limited, and large-scale clinical trials alongside extensive real-world studies are urgently warranted to further validate the long-term efficacy and safety of this approach.

Written informed consent for publication of this case report and accompanying images was obtained from the patient’s legally authorized representative.

## Data Availability

The raw data supporting the conclusions of this article will be made available by the authors, without undue reservation.
